# Functional Recovery After Total Hip Arthroplasty Versus Hemiarthroplasty in Elderly Patients with Femoral Neck Fractures: A Prospective Cohort Study

**DOI:** 10.3390/healthcare14142227

**Published:** 2026-07-22

**Authors:** Djurdjina Bogosavljevic, Goran Tulic, Nikola Bogosavljevic, Kristina Popovic, Snezana Popovac Mijatov, Ivan Selakovic, Sanja Tomanovic Vujadinovic

**Affiliations:** 1 Center for Physical Medicine and Rehabilitation, University Clinical Centre of Serbia, 11000 Belgrade, Serbia; bogosavljevicdjurdjina@gmail.com (D.B.); popovickristina@ymail.com (K.P.); popovacsnezana@gmail.com (S.P.M.); ivan.selakovic@gmail.com (I.S.); 2 Clinic for Orthopedic Surgery and Traumatology, University Clinical Centre of Serbia, 11000 Belgrade, Serbia; tulic05@gmail.com; 3 Faculty of Medicine, University of Belgrade, 11000 Belgrade, Serbia; mf.bg@med.bg.ac.rs; 4 Institute for Orthopedic Surgery “Banjica”, 11000 Belgrade, Serbia

**Keywords:** femoral neck fracture, total hip arthroplasty, hemiarthroplasty, functional recovery, elderly patients, hip fracture rehabilitation, activities of daily living, modified Harris Hip Score, Barthel Index, postoperative outcomes

## Abstract

**Highlights:**

**What are the main findings?**

- Total hip arthroplasty was associated with better postoperative functional recovery than hemiarthroplasty in this prospective cohort of elderly patients with femoral neck fractures.
- Patients treated with total hip arthroplasty achieved higher activities of daily living and hip-specific functional scores throughout postoperative follow-up, including at 6 months postoperatively.

**What are the implications of the main findings?**

- Functional recovery outcomes should be considered alongside traditional surgical outcomes when selecting arthroplasty strategies in geriatric hip fracture management.

**Abstract:**

**Background/Objectives**: Displaced femoral neck fractures in older adults are associated with substantial morbidity, loss of functional independence, and reduced quality of life. Although total hip arthroplasty (THA) and hemiarthroplasty (HA) are widely used surgical options and have been extensively compared, differences in postoperative functional recovery in routine clinical practice continue to be investigated. This study aimed to compare postoperative functional outcomes between THA and HA in elderly patients with femoral neck fractures during postoperative follow-up. **Methods**: This prospective cohort study included 100 patients aged 70–85 years treated surgically for displaced femoral neck fractures from 1 May to 1 December 2024. Patients underwent THA (n = 57) or HA (n = 43). Functional recovery was evaluated using the Barthel Index (BI) and modified Harris Hip Score (mHHS) at 3 weeks, 3 months, and 6 months postoperatively. Changes in functional outcomes during follow-up were analyzed using repeated-measures general linear models adjusted for age, body mass index (BMI), and educational level. Exploratory binary postoperative outcomes were analyzed using logistic regression. **Results**: Patients treated with THA demonstrated significantly better postoperative functional outcomes compared with HA throughout follow-up. In both unadjusted and adjusted analyses, THA patients achieved consistently higher BI and mHHS scores across repeated postoperative assessments, including at 6 months postoperatively. Independent outdoor walking at 3 months postoperatively differed significantly between groups in the unadjusted analysis, although this association did not remain statistically significant after adjustment. In adjusted analyses, patients in the HA group demonstrated significantly higher odds of movement-related hip pain at 6 months postoperatively. **Conclusions**: THA was associated with significantly better postoperative functional outcomes compared with HA in elderly patients with femoral neck fractures. These findings highlight the importance of considering functional recovery outcomes, in addition to traditional surgical outcomes, when selecting arthroplasty strategies in geriatric hip fracture management. Given the observational study design, these findings should be interpreted as associations rather than evidence of a causal treatment effect.

## 1. Introduction

Displaced femoral neck fractures in older adults represent a major public health challenge due to their high incidence, substantial morbidity and mortality, and long-term impact on mobility, functional independence, and quality of life [1,2,3,4]. With global population aging and increasing life expectancy, the incidence of femoral neck fractures and the related healthcare, rehabilitation, and socioeconomic burden are expected to rise substantially in the coming decades [5,6]. Arthroplasty has become the standard treatment option for displaced femoral neck fractures in elderly patients, most commonly performed as hemiarthroplasty (HA) or total hip arthroplasty (THA) [2,3].

Although THA has been associated with improved hip function, mobility, and quality of life in selected patient populations [7,8,9,10], HA remains widely used because of shorter operative time, lower blood loss, and potentially lower dislocation risk [2,4,11]. Consequently, the optimal arthroplasty strategy for active elderly patients with displaced femoral neck fractures remains controversial [9,12,13]. Beyond implant selection, increasing attention has been directed toward postoperative recovery and restoration of functional independence as clinically relevant outcomes in geriatric hip fracture management [1,2,10,14]. This perspective is consistent with the World Health Organization framework for healthy ageing, which emphasizes maintenance of functional ability, mobility, and independence in older adults rather than the absence of disease alone [15,16]. Within this framework, preservation of functional capacity and participation in daily activities are recognized as important determinants of well-being and quality of life in later years [17,18]. In this context, improved postoperative functional recovery following hip arthroplasty may be particularly relevant for elderly patients recovering after femoral neck fractures.

Postoperative recovery after hip fracture surgery is a dynamic process influenced by surgical technique, rehabilitation, and baseline patient characteristics [10,14,19]. Early mobilization and rehabilitation have been associated with improved functional recovery and activities of daily living following hip arthroplasty [1,14]. Comparative outcomes following THA and HA have been extensively investigated in randomized trials, systematic reviews, and registry studies [4,12,13,20]. However, heterogeneity in the collection and reporting of functional outcome measures continues to limit direct comparison between studies and healthcare systems [20,21,22]. The aim of the present prospective cohort study was to compare postoperative functional outcomes between THA and HA in elderly patients with femoral neck fractures in a routine clinical setting using standardized assessments of hip function and activities of daily living during postoperative follow-up.

## 2. Materials and Methods

### 2.1. Study Design and Participants

This prospective cohort study included elderly patients with displaced femoral neck fractures treated surgically at the Clinic for Orthopaedic Surgery and Traumatology, University Clinical Centre of Serbia, and the Institute for Orthopaedic Surgery “Banjica”, Belgrade, Serbia, from 1 May to 1 December 2024. Patients were classified according to the type of arthroplasty received (THA or HA) as part of routine clinical decision-making.

Eligible participants were patients aged 70–85 years who were ambulatory and functionally independent before injury (Barthel Index ≥ 90), with preserved or mildly impaired cognitive status (Short Portable Mental Status Questionnaire [SPMSQ] ≥ 6) and American Society of Anesthesiologists (ASA) score ≤ 3. Exclusion criteria included pathological fractures, polytrauma, terminal illness, fracture duration longer than 4 weeks, and body mass index (BMI) > 30 kg/m^2^.

### 2.2. Surgical Treatment and Rehabilitation

The type of arthroplasty was determined according to routine clinical decision-making and surgeon assessment. Early postoperative rehabilitation was initiated during hospitalization according to a standardized rehabilitation protocol applied to all patients. The rehabilitation program included respiratory and peripheral circulation exercises, range-of-motion exercises for the operated hip, strengthening of hip and thigh musculature, balance training, and progressive mobilization.

Following hospital discharge, patients continued rehabilitation according to routine clinical practice either in specialized rehabilitation facilities or outside institutional settings, including home-based physiotherapy or independently performed exercises at home. The same postoperative rehabilitation recommendations and rehabilitation protocol were applied irrespective of the rehabilitation setting. However, although the prescribed rehabilitation protocol was standardized, patients who underwent institutional rehabilitation were supervised throughout the rehabilitation process, whereas those who continued rehabilitation outside institutional settings were not under continuous clinical supervision. Consequently, adherence to the prescribed rehabilitation program, as well as the quality and intensity of rehabilitation after hospital discharge, could not be comprehensively monitored or objectively verified.

### 2.3. Outcome Measures

Baseline demographic and clinical characteristics included age, sex, marital status, educational level, living arrangements, ASA classification, BMI, cognitive status assessed using the SPMSQ, and comorbidity burden assessed using the Charlson Comorbidity Index (CCI).

Functional recovery was evaluated at 3 weeks, 3 months, and 6 months postoperatively using the Barthel Index (BI) and modified Harris Hip Score (mHHS). Secondary and exploratory postoperative outcomes included walking independence, postoperative pain at rest and during movement, rehabilitation status, and postoperative complications.

### 2.4. Ethical Considerations

The study was conducted in accordance with the Declaration of Helsinki. The ethics application process was initiated before the start of patient recruitment, and the Ethics Committee of the Faculty of Medicine, University of Belgrade, was informed through the submitted study documentation that patient enrolment would commence on 1 May 2024. The formal Ethics Committee decision was subsequently issued by the Ethics Committee of the Faculty of Medicine, University of Belgrade (protocol code 25/IX-9, 16 September 2024). Additional institutional ethical approvals were subsequently obtained from the Ethics Committee of the University Clinical Centre of Serbia (protocol code 1600/44, 13 October 2025) and the Ethics Committee of the Institute for Orthopaedic Surgery “Banjica” (protocol code I-58/31-point 3, 27 November 2025). Written informed consent was obtained from all participants. Prior to participation, all participants received and signed an approved participant information sheet and informed consent form.

### 2.5. Statistical Analysis

Data were analysed using descriptive and inferential statistical methods. The descriptive statistics, including means and standard deviations for numerical variables, and numbers and percentages for categorical variables were used to characterize the study sample. Baseline between-group comparisons were performed using independent-samples *t* tests for continuous variables and χ^2^ or Fisher exact tests for categorical variables, as appropriate.

Continuous functional outcomes, including BI and mHHS scores, were analyzed using repeated-measures general linear models. In the unadjusted analysis, this approach corresponded to a mixed-design repeated-measures ANOVA, with follow-up time as the within-subject factor and prosthesis type as the between-subject factor. The prosthesis type × follow-up time interaction was tested to evaluate whether postoperative recovery trajectories differed between the groups. Because the study groups differed at baseline in age, BMI, and education level, adjusted models additionally included these variables as covariates, corresponding to a mixed-design repeated-measures ANCOVA. Estimated marginal means with 95% confidence intervals were calculated for each prosthesis group at each follow-up time point, and model-based between-group comparisons were performed at each time point. Model assumptions were assessed, including homogeneity of variances. Minor deviations from homogeneity were considered acceptable given the relatively balanced group sizes. Parameters analyzed using this method are presented as unadjusted and adjusted estimated means with corresponding 95% confidence intervals (CIs).

Binary postoperative outcomes were analyzed using separate logistic regression models at each time point. In these models, the dependent variable was the respective binary outcome, coded as binary (yes/no), including independent outdoor walking, independent indoor walking, postoperative complications, hip pain at rest, hip pain during movement, and received rehabilitation. Prosthesis type was the main independent variable and associations with it were reported as odds ratios (ORs) with 95% CIs. Adjusted logistic regression models included age, BMI, and education level as covariates. No imputation of missing data was performed.

No formal a priori sample size calculation was performed because this was a prospective observational cohort study, and all eligible patients who met the predefined eligibility criteria during the study period were included.

All tests were two-sided, and *p* < 0.05 was considered statistically significant. Statistical analyses were performed using IBM SPSS Statistics, version 25.

## 3. Results

### 3.1. Baseline Demographic and Clinical Characteristics

A total of 100 patients were included in the study (57 THA and 43 HA). No participants withdrew from the study after enrolment, and all enrolled patients completed the planned 6-month follow-up for the primary functional outcome assessments. The overall mean age was 77.4 ± 4.6 years (range 70–85), with patients in the HA group being significantly older than those in the THA group (*p* = 0.002). There was no significant difference between groups in terms of sex, ASA classification, cognitive status, or comorbidity burden. Patients in the THA group had a significantly higher BMI and educational level compared to the HA group (*p* = 0.044 and *p* = 0.009, respectively). Detailed baseline demographic and clinical characteristics in total and by prosthesis type are presented in Table 1.

### 3.2. Functional Recovery Outcomes

At 3 weeks postoperatively, patients in the THA group had significantly higher adjusted BI scores compared with the HA group (79.5, 95% CI 73.9–85.2 vs. 63.8, 95% CI 57.7–69.9; *p* = 0.007). Similarly, adjusted mHHS scores were significantly higher in the THA group than in the HA group (60.9, 95% CI 58.5–63.4 vs. 55.6, 95% CI 52.9–58.3; *p* = 0.014).

Repeated-measures general linear models demonstrated significantly better longitudinal functional recovery in the THA group compared with the HA group throughout postoperative follow-up. Both unadjusted and adjusted analyses showed significantly higher BI and mHHS scores in the THA group. In the adjusted analysis, THA patients achieved higher BI scores compared with HA patients, with adjusted mean values of 80.8 versus 64.7 at the early postoperative assessment and 93.1 versus 80.7 during follow-up. Similarly, adjusted mHHS scores consistently favoured the THA group, reaching 61.3 versus 56.4 early postoperatively and 77.5 versus 67.7 at later follow-up assessments (all *p* < 0.050). Detailed unadjusted and adjusted mean values with 95% confidence intervals are presented in Table 2.

### 3.3. Exploratory Postoperative Outcomes

Table 3 presents unadjusted and adjusted logistic regression analyses examining postoperative functional outcomes according to prosthesis type at 3 weeks, 3 months, and 6 months. At 3 weeks, prosthesis type was not significantly associated with postoperative functional outcomes in either the unadjusted or adjusted analyses. At 3 months, patients in the HA group had significantly lower odds of independent outdoor walking compared with the THA group in the unadjusted analysis (*p* = 0.008). However, this association did not remain significant after adjustment for age, BMI, and educational level. No significant associations were observed for independent indoor walking, postoperative complications, hip pain, or rehabilitation status at this time point. At 6 months, the adjusted analysis demonstrated significantly higher odds of hip pain during movement in the HA group compared with the THA group (*p* = 0.037). No other significant associations were identified. Wide confidence intervals observed in several models likely reflect the low number of events for some outcomes and limited statistical power and should therefore be interpreted with caution (Table 3).

## 4. Discussion

The present prospective cohort study evaluated postoperative functional recovery following THA and HA in elderly patients with displaced femoral neck fractures. The main finding of this study was that patients treated with THA achieved significantly better postoperative functional outcomes compared with HA throughout follow-up, particularly regarding activities of daily living and hip-specific functional outcomes assessed using the BI and mHHS. In both unadjusted and adjusted analyses, patients treated with THA demonstrated consistently higher functional scores across repeated postoperative assessments, including at 6 months postoperatively. These findings are generally consistent with previous randomized trials, registry studies, and meta-analyses reporting improved functional outcomes and quality of life following THA in selected elderly patients with femoral neck fractures [1,2,7,8,9].

Several previous studies have demonstrated that THA may provide improved postoperative mobility, hip function, and patient-reported outcomes compared with HA, despite the potentially greater surgical burden associated with the procedure [1,2,8,9,10,21].

In contrast, HA has traditionally been associated with shorter operative time, lower blood loss, and potentially lower dislocation risk, contributing to the ongoing controversy regarding the optimal arthroplasty strategy in elderly patients [11,23,24]. Previous meta-analyses have additionally suggested that although THA may offer improved functional outcomes and quality of life, these advantages should be interpreted alongside the potentially greater surgical complexity and risk of dislocation-related complications [9,12,13].

The present study prospectively evaluated postoperative functional recovery following THA and HA in elderly patients with femoral neck fractures using repeated assessments of activities of daily living and hip-specific function during postoperative follow-up. Comparative outcomes following THA and HA have been extensively investigated in randomized trials, systematic reviews, and registry studies, and the present findings should be interpreted within the context of this existing body of evidence [4,12,13,20].

In the present cohort, patients treated with THA demonstrated significantly better postoperative functional outcomes throughout follow-up, including at 3 weeks, 3 months, and 6 months postoperatively. The adjusted between-group difference in the Barthel Index at 6 months was approximately 10 points. Because the Barthel Index reflects independence in activities of daily living, this difference may indicate better functional independence in elderly patients recovering from femoral neck fractures [10,14]. However, the clinical importance of this difference should be interpreted cautiously because no established threshold for a clinically meaningful between-group difference in this population has been defined. Furthermore, given the observational design of the present study and the possibility of residual confounding, these findings should not be considered evidence of a causal treatment effect. From a clinical perspective, improved functional recovery after THA may facilitate earlier restoration of mobility and greater independence in activities of daily living, outcomes that are particularly relevant in elderly hip fracture patients because delayed recovery is associated with frailty, institutionalization, and reduced quality of life [1,10,14]. Earlier recovery of mobility and activities of daily living may influence rehabilitation intensity, mobility goals, and expectations regarding functional recovery during postoperative follow-up [25]. In geriatric patients, prolonged immobility may rapidly contribute to muscle weakness, physical deconditioning, frailty, and loss of independence, highlighting the clinical importance of early postoperative functional recovery.

In addition to global functional recovery scores, the adjusted analysis demonstrated significantly higher odds of movement-related hip pain in the HA group at 6 months postoperatively [10,14]. Furthermore, independent outdoor walking at 3 months postoperatively differed significantly between groups in the unadjusted analysis, although this association did not remain statistically significant after adjustment. These findings suggest that postoperative recovery following hip fracture surgery may differ according to arthroplasty type not only in terms of hip-specific function, but also regarding restoration of mobility and movement-related postoperative pain. Similar advantages of THA regarding postoperative mobility and patient-reported recovery have been described in previous cohort studies and randomized trials [7,8,10]. Given the relatively small number of events for several binary outcomes, these findings should be interpreted cautiously.

From a clinical perspective, lower levels of movement-related postoperative pain may be particularly relevant in elderly patients recovering after hip fracture surgery. Persistent pain may limit mobility, reduce participation in rehabilitation, and delay recovery of functional independence [26,27]. Therefore, lower postoperative pain after THA may represent an additional clinically relevant factor that could facilitate rehabilitation and postoperative functional recovery in this vulnerable patient population.

As treatment allocation reflected routine clinical practice rather than random assignment, baseline differences between the treatment groups were presented and were addressed by statistical adjustment for age, BMI, and educational level. Similar challenges related to treatment selection and potential selection bias have been recognized in previous observational studies comparing THA and HA for femoral neck fractures [1,11].

The present study has several limitations. First, the study was not randomized, and treatment allocation was based on routine clinical decision-making. Although strict inclusion and exclusion criteria contributed to cohort homogenization and reduction in baseline clinical heterogeneity, residual confounding cannot be completely excluded. Furthermore, because treatment allocation reflected routine clinical practice rather than random assignment, selection bias is inherent to the study design despite adjustment for measured baseline differences. In addition, although the analyses were adjusted for age, BMI, and educational level, several potentially relevant factors known to influence postoperative functional recovery were not comprehensively assessed, including pre-fracture functional status, frailty, nutritional status, sarcopenia, cognitive function, socioeconomic factors, social support, and adherence to postoperative rehabilitation [1,21].

Although standardized rehabilitation was provided during hospitalization, adherence to rehabilitation after hospital discharge could not be systematically monitored or objectively quantified. Furthermore, rehabilitation delivered outside officially registered rehabilitation centres was not consistently documented, which may have contributed to residual confounding when interpreting the functional outcomes. In addition, although operative variables such as the surgical approach, implant type and fixation method, operative duration, and intraoperative blood loss were recorded as part of routine clinical documentation, they were not included in the predefined analyses because the primary objective of the present study was to evaluate postoperative functional recovery rather than operative characteristics. Surgeon experience was not systematically recorded. Consequently, the potential influence of these variables on postoperative functional recovery could not be evaluated in the present study. Residual confounding due to these factors should therefore be considered when interpreting the study findings. Accordingly, the observed associations between prosthesis type and postoperative functional recovery should be interpreted as observational and should not be considered evidence of causality.

Second, the study was conducted at two orthopaedic centres, and subtle differences in surgical decision-making or postoperative management may have influenced outcomes despite the use of standardized in-hospital rehabilitation protocols.

Third, the sample size was modest. No formal a priori sample size calculation was performed because this was a prospective observational cohort study in which all eligible patients who met the predefined eligibility criteria during the study period were included. Consequently, statistical power may have been limited for some secondary and exploratory outcomes.

Fourth, although no participants withdrew from the study, the findings should primarily be interpreted at the group level because the study was designed to evaluate postoperative functional recovery in an observational cohort rather than individual recovery trajectories. Therefore, postoperative functional recovery should primarily be interpreted at the group level across follow-up assessments rather than at the individual patient level.

Finally, the follow-up period was limited to six months and was not intended to evaluate long-term outcomes such as implant survival, revision surgery, or late complications, which require longer follow-up than the present study was designed to provide [4,13,28]. Future prospective studies with larger cohorts and longer follow-up are warranted to further evaluate postoperative functional recovery and long-term outcomes following THA and HA in elderly patients with femoral neck fractures.

Despite these limitations, the study also has important strengths, including its prospective design, relatively homogeneous cohort achieved through strict eligibility criteria, standardized early rehabilitation approach, clinically relevant follow-up intervals, and comprehensive assessment of postoperative functional recovery using activities of daily living and hip-specific functional measures.

## 5. Conclusions

In conclusion, THA was associated with significantly better postoperative functional outcomes compared with HA in elderly patients with femoral neck fractures. Patients treated with THA demonstrated consistently higher activities of daily living and hip-specific functional scores throughout follow-up, including at 6 months postoperatively. In addition, exploratory postoperative outcomes suggested more favourable mobility-related recovery following THA, while adjusted analyses demonstrated higher odds of movement-related postoperative pain in the HA group at 6 months. These findings highlight the importance of considering functional recovery outcomes, in addition to traditional surgical outcomes, when selecting arthroplasty strategies in geriatric hip fracture management. However, given the observational nature of the present study and the potential for residual confounding, the observed associations should be interpreted with caution and should not be considered evidence of a causal treatment effect.

## Figures and Tables

**Table 1:** Baseline demographic and clinical characteristics by prosthesis type.

Variables	Total n (%) = 100	Surgery		*p*
		THA n (%) = 57	HA n (%) = 43	
Sex				0.537
Male	16 (16.0)	8 (14.0)	8 (18.6)	
Female	84 (84.0)	49 (86.0)	35 (81.4)	
Age, mean ± SD	77.4 ± 4.6	76.2 ± 4.5	79.0 ± 4.2	0.002
Marital status				0.610
Single	61 (61.0)	36 (63.2)	25 (58.1)	
Married	39 (39.0)	21 (36.8)	18 (41.9)	
Education level				0.009
Higher	20 (20.0)	15 (26.3)	5 (11.6)	
High school	41 (41.0)	27 (47.4)	14 (32.6)	
Elementary	39 (39.0)	15 (26.3)	24 (55.8)	
Living arrangements				0.143
Living alone	36 (36.0)	24 (42.1)	12 (27.9)	
Living with someone	64 (64.0)	33 (57.9)	31 (72.1)	
ASA				0.481
Low (mild systemic disease)	17 (17.0)	11 (19.3)	6 (14.0)	
Moderate to severe	83 (83.0)	46 (80.7)	37 (86.0)	
BMI, mean ± SD	24.4 ± 3.4	25.0 ± 3.1	23.7 ± 3.5	0.044
SPMSQ				1.000
Intact cognitive function	93 (93.0)	53 (93.0)	40 (93.0)	
Mild cognitive impairment	7 (7.0)	4 (7.0)	3 (7.0)	
CCI				0.229
Moderate risk (CCI 3–4)	56 (56.0)	35 (61.4)	21 (48.8)	
High risk (CCI ≥ 5)	44 (44.0)	22 (38.6)	22 (51.2)	

Data are presented as n (%). ASA = American Society of Anesthesiologists physical status classification; BMI = body mass index; SPMSQ = Short Portable Mental Status Questionnaire; CCI = Charlson Comorbidity Index.

**Table 2:** Functional outcomes according to prosthesis type during follow-up.

	Unadjusted						
	3 weeks		3 months		6 months		*p*
	THA	HA	THA	HA	THA	HA	
BI (n = 83)	79.9 (74.2–85.6)	64.4 (57.8–71.1)	92.2 (87.0–97.4)	80.9 (74.8–86.9)	94.3 (89.1–99.4)	85.4 (79.4–91.5)	0.002
mHHS (n = 82)	60.7 (58.2–63.2)	56.8 (53.8–59.8)	76.4 (72.5–80.3)	68.3 (63.6–72.9)	86.0 (82.2–89.9)	77.4 (72.8–81.9)	0.004
	Adjusted						
	3 weeks		3 months		6 months		*p*
	THA	HA	THA	HA	THA	HA	
BI (n = 83)	80.8 (75.0–86.5)	64.7 (58.4–71.0)	93.1 (87.7–98.4)	80.7 (74.8–86.5)	95.2 (89.9–100.5)	85.1 (79.3–90.9)	0.002
mHHS (n = 82)	61.3 (58.7–63.9)	56.4 (53.6–59.2)	77.5 (73.5–81.4)	67.7 (63.3–72.1)	87.2 (83.3–91.0)	76.8 (72.6–81.1)	<0.001

Adjusted for age, BMI and education. Data presented as means and 95% CI. BI = Barthel Index; mHHS = modified Harris Hip Score.

**Table 3:** Logistic regression analysis of postoperative functional outcomes according to prosthesis type at 3 weeks, 3 months, and 6 months.

	THA n (%)	HA n (%)	Unadjusted		Adjusted	
3 weeks	n = 56	n = 42	OR (95% CI)	*p*	OR (95% CI)	*p*
Independent outdoor walking	9 (16.1)	6 (14.3)	0.87 (0.28–2.67)	0.808	0.85 (0.24–2.96)	0.797
Independent indoor walking	53 (94.6)	38 (90.5)	0.54 (0.11–2.54)	0.434	0.49 (0.09–2.85)	0.431
Complications	3 (5.6)	3 (7.3)	1.34 (0.26–7.02)	0.727	2.12 (0.32–14.15)	0.437
Hip pain at rest	6 (11.1)	3 (7.5)	0.65 (0.15–2.77)	0.559	0.35 (0.06–1.92)	0.228
Hip pain during movement	17 (31.5)	17 (42.5)	1.61 (0.69–3.76)	0.273	1.65 (0.63–4.31)	0.307
Rehabilitation received	28 (50.0)	22 (55.0)	1.22 (0.54–2.76)	0.629	0.63 (0.24–1.70)	0.366
3 months	n = 52	n = 39				
Independent outdoor walking	44 (84.6)	23 (59.0)	0.26 (0.10–0.70)	0.008	0.38 (0.13–1.13)	0.083
Independent indoor walking	50 (96.2)	36 (92.3)	0.48 (0.08–3.02)	0.434	0.70 (0.09–5.69)	0.736
Complications	6 (11.3)	5 (12.5)	1.12 (0.32–3.96)	0.862	0.85 (0.19–3.73)	0.825
Hip pain at rest	8 (15.7)	3 (7.9)	0.46 (0.11–1.87)	0.278	0.42 (0.09–1.96)	0.270
Hip pain during movement	10 (19.6)	9 (23.7)	1.27 (0.46–3.52)	0.643	1.87 (0.57–6.07)	0.300
Rehabilitation received	29 (55.8)	21 (53.8)	0.93 (0.40–2.13)	0.855	0.65 (0.24–1.79)	0.404
6 months	n = 51	n = 38				
Independent outdoor walking	43 (84.3)	27 (71.1)	0.46 (0.16–1.28)	0.136	0.65 (0.20–2.11)	0.470
Independent indoor walking	47 (92.2)	36 (94.7)	1.53 (0.27–8.83)	0.633	1.37 (0.19–10.02)	0.756
Complications	2 (3.9)	1 (2.6)	0.67 (0.06–7.58)	0.740	0.28 (0.01–6.34)	0.425
Hip pain at rest	1 (2.0)	2 (5.3)	2.67 (0.23–30.57)	0.431	5.89 (0.34–102.88)	0.225
Hip pain during movement	4 (8.2)	6 (15.8)	2.11 (0.55–8.09)	0.276	6.63 (1.12–39.33)	0.037
Rehabilitation received	31 (60.8)	25 (65.8)	1.24 (0.52–2.98)	0.629	0.83 (0.29–2.35)	0.728

Adjusted models were controlled for age, BMI, and educational level. Wide CIs in some models reflect the limited number of outcome events.

## Data Availability

The anonymized minimal dataset supporting the findings of this study is available from the corresponding author upon reasonable request. Individual-level clinical data are not publicly available due to ethical restrictions and the need to protect participant privacy and confidentiality.

## Abbreviations

The following abbreviations are used in this manuscript:

ASA	American Society of Anesthesiologists
BI	Barthel Index
BMI	Body Mass Index
CCI	Charlson Comorbidity Index
CI	Confidence Interval
GLM	General Linear Model
HA	Hemiarthroplasty
mHHS	Modified Harris Hip Score
OR	Odds Ratio
SD	Standard Deviation
SPMSQ	Short Portable Mental Status Questionnaire
THA	Total Hip Arthroplasty
